# Substrate Directed Regioselective Monobromination of Aralkyl Ketones Using *N*-Bromosuccinimide Catalysed by Active Aluminium Oxide: ****α****-Bromination versus Ring Bromination

**DOI:** 10.1155/2014/751298

**Published:** 2014-03-04

**Authors:** Reddy Bodireddy Mohan, G. Trivikram Reddy, N. C. Gangi Reddy

**Affiliations:** Department of Chemistry, School of Physical Sciences, Yogi Vemana University, Kadapa, Andhra Pradesh 516 003, India

## Abstract

Bromination of aralkyl ketones using *N*-bromosuccinimide in presence of active Al_2_O_3_ provided either **α**-monobrominated products in methanol at reflux or mononuclear brominated products in acetonitrile at reflux temperature with excellent isolated yields depending on the nature of substrate employed. The **α**-bromination was an exclusive process when aralkyl ketones containing moderate activating/deactivating groups were subjected to bromination under acidic Al_2_O_3_ conditions in methanol at reflux while nuclear functionalization was predominant when aralkyl ketones containing high activating groups were utilized for bromination in presence of neutral Al_2_O_3_ conditions in acetonitrile at reflux temperature. In addition, easy isolation of products, use of inexpensive catalyst, short reaction time (10–20 min), and safe operational practice are the major benefits in the present protocol.

## 1. Introduction

Nowadays, researchers are focusing on the development of more acceptable bromination protocols to accomplish increasing demands for “organohalogen” chemistry and to achieve higher efficiency and selectivity of the bromination reactions which include *α*-bromination and nuclear bromination. The resulting *α*-brominated or nuclear brominated products acquired wide range of utility in organic synthesis [1–3]. Nuclear brominated ketones are found to be useful intermediates in C–C coupling reactions, as precursors to organometallic species and in nucleophilic substitutions.

It is well known that use of molecular bromine [4] as a basic electrophilic brominating reagent has several drawbacks. Alternative reagents were reported in the literature, for example, cupric bromide [5], dioxane dibromide [6], *tetra*butyl ammonium tribromide [7], H_2_O_2_-HBr [8], bromodimethyl sulfoniumbromide [9], ethylene bis(*N*-methyl imidazolium) ditribromide [10], trihaloisocyanuric acids [11], pyridinium bromochromate [12], and NH_4_Br-oxone [13]. In addition, a popular and superior brominating agent such as *N*-bromosuccinimide [14] was utilized for *α*-bromination of carbonyl compounds using a radical initiator such as azobisisobutyronitrile (AIBN) or dibenzoyl peroxide (BPO) [15] and, later, it has been demonstrated that the reactivity of NBS could be modulated with ionic liquids [16], photochemical energy [17]; sonochemical energy [18], solvent free reaction conditions (SFRC) [19] and various catalysts such as Mg(ClO_4_)_2_ [20], NH_4_OAc [21], amberlyst-15 [22], silica supported NaHCO_3_ [23], sulfonic acid functionalized silica [24], FeCl_3_ [25], montmorillonite-K10 [26], and Silica gel [27]. NBS also is utilized for ring or nuclear bromination using various catalysts such as H_2_SO_4_-CF_3_CO_2_H [28], *p*-toluenesulfonic acid [29], dibromodimethylhydantoin in aqueous base [30], amberlyst [31], and HZSM-5 [32].

**Scheme 1:**
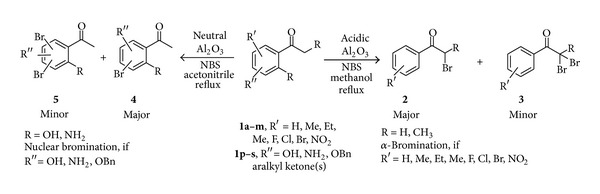
Substrate directed regioselective synthesis of monobrominated aralkyl ketones.

It is well recognized that solids play a significant role in the development of cleaner technologies through their abilities to act as catalysts, support reagents, entrain by-products, and influence product selectivity, and several books on the applications of solids in organic synthesis have appeared [33–35]. However, the catalysts and any accompanying reagent used along with *N*-bromosuccinimide should be easily available to develop simple and efficient bromination procedure. Alumina (Al_2_O_3_) has been used as a catalyst [36] in a wide variety of industrial processes for many years [37]. But Alumina (Al_2_O_3_) has limited catalytic applications in synthetic organic chemistry [38]. However, on deep investigation it is found that aralkyl ketones with moderate activating/moderate or high deactivating groups undergo exclusively *α*-bromination in the presence of acidic alumina in methanol, while substrates with high activating groups undergo nuclear bromination predominantly in the presence of neutral alumina in acetonitrile at reflux temperature, as shown in Scheme 1

**Scheme 2:**
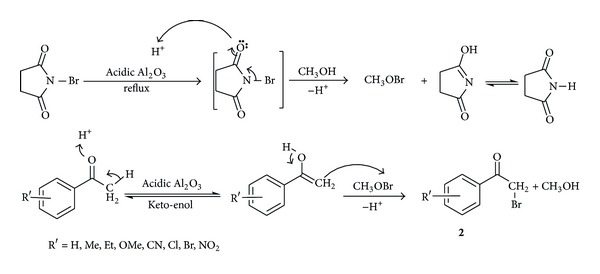
Plausible mechanism for *α*-bromination.

Plausible mechanism for bromination of aralkyl ketones containing moderate activating/deactivating groups may be involved in the exclusive formation of enolic form of ketone in presence of acidic Al_2_O_3_ aids predominant formation of *α*-brominated product (**2**). In addition, acidic Al_2_O_3_ may enhance the rate of release of bromonium ion from NBS and subsequent capture of bromonium ion by nucleophilic solvent such as methanol leads to rapid completion of the reaction (10–15 min) as shown in Scheme 2.

**Scheme 3:**
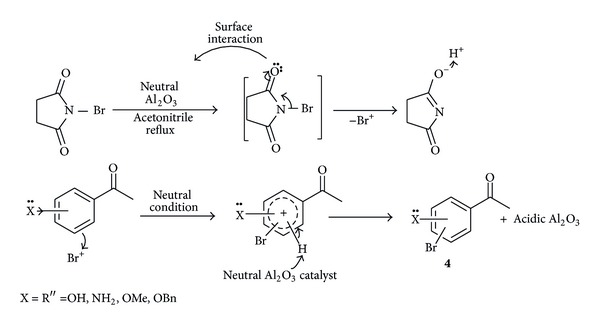
Plausible mechanism for nuclear functionalization.

In contrast, aralkylketones containing high electron donating groups may be susceptible to rapid activation of the aromatic ring of substrate and rapid release of bromonium ion from NBS *via* surface interaction with neutral Al_2_O_3_ which leads to exclusive formation of nuclear brominated product (**4**) in acetonitrile based on substrate employed and the catalyst may assist in abstracting the proton during the course of bromination as shown in Scheme 3.

## 2. Results and Discussion

Initially, two types of substrates were selected for optimization of reaction conditions using *N*-bromosuccinimide in presence of either acidic or neutral Al_2_O_3._ Accordingly, acetophenone and 4′-hydroxy acetophenone were utilized for the evaluation of reaction conditions such as effect of solvent, temperature, catalyst, and catalyst load and the obtained results are discussed below.

The effect of catalyst on the course of bromination of acetophenone was studied and the obtained results were summarized in Table 1. It was observed that acidic Al_2_O_3_ (entry 3, Table 1) provided better yields of desired *α*-brominated product (**2a**) compared to neutral Al_2_O_3_ (entry 5) in methanol at reflux temperature. It might be attributed that acidic nature of Al_2_O_3_ may enhance the formation of enol form of substrate and subsequent bromination also. In addition, acidic nature of catalyst might boost the rate of release of bromonium ion from *N*-bromosuccinimide followed by the capture of bromonium ion by nucleophilic solvent such as methanol. Reuse of acidic Al_2_O_3_ provided 89%, 86%, 81%, and 72% yields of product (**2a**) for first, second, third, and fourth time, respectively.

We also studied the effect of acidic Al_2_O_3_ catalyst load on the course of *α*-bromination. Towards this direction, 5%, 10%, and 15% of catalyst (w/w) was applied and the obtained yields of product **2a **were 71%, 89%, and 78%, respectively. The study revealed that 10% (w/w) of acidic Al_2_O_3_ is optimum for better isolated yield of product **2a**.

Later, we focused on the optimization of other reaction parameters such as effect of solvent in presence of 10% of acidic Al_2_O_3_. Consequently, the effect of solvents on the course of *α*-bromination was studied using different types of solvents and the obtained results were presented in Table 2. For example MeOH, EtOH, H_2_O, CH_3_CN, THF, CH_2_Cl_2_, are CHCl_3_ were provided 89%, 74%, 15%, 51%, 56%, 44%, 55%, and 48% yields of desired product **2a**, respectively. Interestingly, a high yield (89%) of product **2a** was obtained when methanol was used as solvent (entry 1, Table 1).

As evident from the literature [26], portion wise addition of *N*-bromosuccinimide caused to control the release of bromonium ion and it provided improved yields of *α*-brominated product (**2a**). The same was implemented in the present investigation and *N*-bromosuccinimide was added portion wise (10 portions). As a consequence, the isolated yield of product **2a **was improved up to 89% compared to one time NBS addition, which provided lower yield (74%) of product **2a**.

With the help of optimized reaction conditions, further scope and generality of the *α*-bromination was tested by utilizing a series of aralkyl ketones containing moderate activating/deactivating groups and highly deactivating groups in presence of acidic Al_2_O_3_ in methanol and the obtained results were presented in Table 3. It was found that presence of moderate activating and deactivating groups on phenyl ring of aralkylketones favors the *α*-bromination (entries 1–10 and 11, Table 3 and entries 1 and 2, Table 4) with excellent isolated yields of desired product(s), whilst presence of highly deactivating groups provided lower yields of product (entries 12 and 13, Table 3). Fascinatingly, *α*-brominated product (**2k**) was formed exclusively even though 4′-methoxyacetophenone contains high activating group (-OMe) (entry 11, Table 3).

Acenaphthones(s) were subjected to bromination using *N*-bromosuccinimide in presence of acidic Al_2_O_3_ in methanol at reflux temperature and they provided respective *α*-brominated products (**2n** and **2o**, entries 1 and 2, Table 4) exclusively. The obtained results were presented in Table 4.

In continuation of our agenda to explore the effect of Alumina catalyst on different types of substrates, we focused on the effect of neutral alumina on the course of bromination. As a result, we selected 4′-hydroxy acetophenone (**1p**) as model substrate and the reaction conditions were optimized with respect to it. The obtained results were presented in Table 5.

When we applied neutral Al_2_O_3_ (entry 6, Table 5) for bromination reaction of 4′-hydroxy acetophenone (**1p**), good yield of nuclear brominated product (**4a**) was obtained compared to acidic Al_2_O_3_ (entry 4, Table 5) in methanol at reflux temperature. It may be attributed that acidic Al_2_O_3_ might deactivate the electron donating efficiency of high activating groups. In contrast, neutral Al_2_O_3_ might facilitate the activation of the aromatic ring of aralkyl ketone as well as the rate of formation of bromonium ion from NBS *via* surface interaction with reactants.

To improve the yields of product **4a **further, we have studied the effect of solvent on the course of bromination and the obtained results were depicted in Table 6. Solvents such as MeOH, EtOH, H_2_O, CH_3_CN, THF, CH_2_Cl_2_, and CHCl_3_ were provided 86%, 61%, 22%, 94%, 48%, 40%, 30%, and 34% yields of desired product **4a**, respectively. The solvent study disclosed that acetonitrile is the best to obtain maximum yield (94%) of the product **4a** (entry 4, Table 6) compared to methanol (86%) (entry 1, Table 6). Reuse of catalyst provided 94%, 89%, 80%, and 74% yields of product (**4a**) for first, second, third, and fourth time, respectively.

It was observed that portion wise (10 portions) addition of *N*-bromosuccinimide provided improved yield (94%) of product **4a** compared to one-time addition of *N*-bromosuccinimide which provided lower yield (65%) of product **4a**. The study revealed that the portion wise addition of NBS resulted in improved yields of product **4a**.

With the help of optimized reaction conditions further generality of the nuclear bromination was tested with a variety of substrates (**1p**–**r**) containing high activating groups in presence of neutral Al_2_O_3_ in acetonitrile at reflux temperature and the obtained results were summarized in Table 7. It was found that neutral Al_2_O_3_ provided good yields of nuclear monobrominated products (entries 1–5, Table 7).

Excess use of *N*-bromosuccinimide (24 mmol) provided excellent yields of nuclear dibrominated products **5a** and **5b** (entries 6 and 7, Table 7). The 4′-methoxy acetophenone (**1k**) in acetonitrile solvent in presence of neutral Al_2_O_3_ provided exclusively nuclear brominated product **5e** instead of **2k**.

Encouraged by the above fruitful results, we attempted to prepare 1-(3,5-bis(benzyloxy) phenyl)-2-bromoethanone (**2p**), a key *α*-brominated intermediate product in the synthesis of terbutaline sulphate drug starting from the substrate, 3′,5′-dibenzyloxy acetophenone (**1s**).

**Scheme 4:**
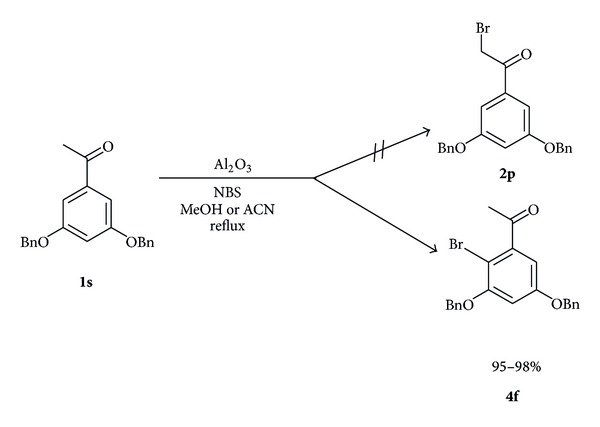
Nuclear bromination of 3,5-dibenzyloxy acetophenone (**1s**).

Interestingly, we obtained 95–98% yields of mononuclear brominated product of 1-(3,5-bis(benzyloxy)-4-bromophenyl)ethanone (**4f**) exclusively both in presence of acidic Al_2_O_3_ in methanol and neutral Al_2_O_3_ in acetonitrile as depicted in Scheme 4.

## 3. Conclusion

In summation, we demonstrated that using Al_2_O_3_ as catalyst, monobromination of various aralkyl ketones was achieved in high yield with high substrate directed regioselectivity (*α*-bromination versus nuclear bromination) using NBS. The *α*-bromination was the exclusive process when aralkyl ketones containing moderate activating/deactivating groups were subjected to bromination under acidic Al_2_O_3_ conditions in methanol at reflux while nuclear functionalization was predominant when aralkyl ketones containing high activating groups were utilized for bromination in presence of neutral Al_2_O_3_ conditions in acetonitrile at reflux temperature. Thus, our new protocol offers safe operational procedure, short reaction time (10–20 min), and use of inexpensive and recyclable catalyst for 3 times without loss of activity.

## 4. Experimental

### 4.1. General

All chemicals used were reagent grade and were used as received without further purification. Ketones were purchased from Acros, Merck, and SD Fine Chemicals Ltd., Mumbai, India and Avra Laboratories Ltd., Hyderabad, India. *N*-bromosuccinimide was purchased from Merck. Methanol (99.0%) was purchased from SD Fine Chemicals Ltd. EtOAc was purchased from Merck. The double distilled millipore deionized water was used for work up. Melting points were determined in open capillaries on REMI melting point apparatus and were uncorrected. ^1^H NMR spectra were recorded on a Varian 400 MHz. Chemical shifts were expressed in parts per million (ppm). Splitting patterns describe apparent multiplicities and are designated as s (singlet), d (doublet), t (triplet), q (quartet), m (multiplet), or br (broad). Mass spectra (MS) are acquired on agilent, model-6410, Triple Quad LC MS. Thin-layer chromatography was performed on 0.25 mm Merck silica gel plates (60F-254) and visualized with UV light. Column chromatography was performed on silica gel (finer than 200 mesh, Merck).

### 4.2. Active Al_**2**_O_**3**_ Catalyst Specification(s)

#### 4.2.1. Acidic Al_**2**_O_**3**_

Acidic Al_2_O_3_ is commercially available (Merck made) and has the following characteristics (i) White crystalline solid, (ii) pH value (10% aqueous suspension) is 4-5; (iii) active according to Brockmann for column chromatography.

#### 4.2.2. Neutral Al_**2**_O_**3**_

Neutral Al_2_O_3_ is commercially available (Fisher made) and has the following characteristics (i) White crystalline solid, (ii) pH value (10% aqueous suspension) is 6.8–7.8; (iii) active according to Brockmann for column chromatography.

### 4.3. General Experimental Procedure for *α*-Bromination

In a 100 mL RB flask fitted with condenser, the aralkylketone **1a** (10 mmol), 10% (w/w) active Al_2_O_3_ catalyst, and methanol (20 vol) were added. The temperature of the reaction mass was raised to reflux. Then, *N*-bromosuccinimide (12 mmol) was added portion wise (10 portions). After completion of the reaction, as monitored by TLC (mobile phase is a mixture of 5 mL n-hexane and 3 drops of EtOAc; Caution: there is need to run TLC for 3–5 times for clear distinction between mono versus dibrominated products), the reaction mixture was filtered to collect the catalyst and the solvent was removed under reduced pressure. Water (100 mL) was added to the reaction mass and the formed *α*-brominated product was extracted thrice with EtOAc (3 × 50 mL). Layers were separated and the organic layer was collected and then washed thrice with water (3 × 50 mL). The organic layer was collected and dried over anhydrous Na_2_SO_4_ and the solvent was removed using rotary evaporator. Pure *α*-brominated product (**2a**) was obtained from crude residue after purification by column chromatography over silica gel using a mixture of n-hexane and EtOAc (99 : 1 ratio).

### 4.4. General Experimental Procedure for Nuclear Bromination

In a 100 mL RB flask fitted with condenser, the aralkylketone **1p** (10 mmol), 10% (w/w) neutral Al_2_O_3_ catalyst, and acetonitrile (20 vol) were added. The temperature of the reaction mass was raised to reflux. Then, *N*-bromosuccinimide (12 mmol) was added portion wise (10 portions). After completion of the reaction, as monitored by TLC (mobile phase is a mixture of 5 mL n-hexane and 3 drops of EtOAc; caution: there is need to run TLC for 3–5 times for clear distinction between mono- versus dibrominated products), the reaction mixture was filtered to collect the catalyst and the solvent was removed under vacuum. Water (100 mL) was added to the reaction mass and the formed nuclear brominated product was extracted thrice with EtOAc (3 × 50 mL). Layers were separated and the organic layer was collected and it was washed thrice with water (3 × 50 mL). The organic layer was collected and dried over anhydrous Na_2_SO_4_ and the solvent was removed using rotary evaporator. Pure nuclear brominated product (**4a**) was obtained from crude residue after purification by column chromatography over silica gel using a mixture of n-hexane and EtOAc (99 : 1 ratio).

### 4.5. Physical and Spectral Characterization Data

#### 4.5.1. 2-Bromo-1-phenyl Ethanone (**2a**)

Off-white solid, yield: 89%; m.p. 48–50°C; FT-IR (KBr, cm^−1^): 3085.6, 2999.6, 1694.3, 1589.7, 1485.8, 1283.6, 1198.8, 811.8, 665.7, 548.1. ^1^H-NMR (400 MHz, CDCl_3_, *δ*/ppm): 8.00–8.10 (2H, m, arom H), 7.43–7.78 (3H, m, arom H), 4.50 (2H, s, –CH_2_). MS (ESI) *m/z* 199.05 [M^+∙^+H, ^79^Br], 201.1 [M^+∙^+H+2, ^81^Br].

#### 4.5.2. 2-Bromo-1-phenylpropan-1-one (**2b**)

Colorless liquid, yield: 74%; b.p. 247–251°C; ^1^H-NMR (400 MHz, CDCl_3_, *δ*/ppm): 8.10 (2H, d, *J* = 7.2 Hz, arom H), 7.72–7.64 (1H, m, arom H), 7.50−7.60 (2H, m, arom H), 5.30 (1H, q, *J* = 6.8 Hz, alkyl H), 2.10 (3H, d, *J* = 7.2 Hz, –CH_3_); MS (ESI) *m/z* 213.03 [M^+∙^+H, ^79^Br], 215.2 [M^+∙^+H+2, ^81^Br].

#### 4.5.3. 2-Bromo-1-o-tolylethanone (**2c**)

Colorless liquid, yield: 86%; b.p. 81–83°C; ^1^H-NMR (400 MHz, CDCl_3_, *δ*/ppm): 8.10 (1H, d, arom H, *J* = 8.0 Hz), 7.70 (1H, t, *J* = 7.6 Hz, arom H), 7.40 (1H, t, *J* = 7.2 Hz, arom H), 7.10 (1H, d, *J* = 8.0 Hz, arom H), 4.50 (2H, s, –CH_2_), 2.50 (3H, s, –CH_3_); MS (ESI): *m/z* 212.96 [M^+∙^+H, ^79^Br], 214.97 [M^+∙^+H+2, ^81^Br].

#### 4.5.4. 2-Bromo-1-m-tolylethanone (**2d**)

Colorless liquid, yield: 88%; b.p. 234°C; ^1^H-NMR (400 MHz, CDCl_3_, *δ*/ppm): 7.83 (1H, d, *J* = 8.0 Hz, arom H), 7.58 (1H, s, arom H), 7.35 (1H, d, *J* = 7.6 Hz, arom H), 7.10 (1H, t, *J* = 7.2 Hz, arom H), 4.60 (2H, s, –CH_2_), 2.48 (3H, s, –CH_3_); MS (ESI): *m/z* 213.15 [M^+∙^+H, ^79^Br], 215.1 [M^+∙^+H+2, ^81^Br].

#### 4.5.5. 2-Bromo-1-p-tolylethanone (**2e**)

Off-white solid, yield: 90%, m.p. 51–53°C. ^1^H-NMR (400 MHz, CDCl_3_, *δ*/ppm): 7.65 (2H, d, *J* = 7.6 Hz, arom H), 7.00 (2H, d, *J* = 7.2 Hz, arom H), 4.70 (2H, s, –CH_2_), 2.54 (3H, s, –CH_3_); MS (ESI): *m/z* 212.89 [M^+∙^+H, ^79^Br], 214.81 [M^+∙^+H+2, ^81^Br].

#### 4.5.6. 2-Bromo-1-(4-ethylphenyl)ethanone (**2f**)

Colorless liquid, yield: 85%; b.p. 288.5°C. ^1^H-NMR (400 MHz, CDCl_3_, *δ*/ppm): 7.45 (2H, d, *J* = 8.0 Hz, arom H), 7.20 (2H, d, *J* = 7.6 Hz, arom H), 4.60 (2H, s, –CH_2_), 2.64 (2H, q, *J* = 7.6 Hz, –CH_2_), 1.54 (3H, t, *J* = 6.8 Hz, –CH_3_); MS (ESI): *m/z* 226.95 [M^+∙^+H, ^79^Br], 229.08 [M^+∙^+H+2, ^81^Br].

#### 4.5.7. 4-(2-Bromoacetyl)benzonitrile (**2g**)

Off-white solid, yield: 73%, m.p. 90–92°C. ^1^H-NMR (400 MHz, CDCl_3_, *δ*/ppm): 8.24 (2H, d, *J* = 7.2 Hz, arom H), 7.91 (2H, d, *J* = 7.6 Hz, arom H), 4.32 (2H, s, –CH_2_); MS (ESI): *m/z* 224.15 [M^+∙^+H, ^79^Br], 226.01 [M^+∙^+H+2, ^81^Br].

#### 4.5.8. 2-Bromo-1-(3-chlorophenyl)ethanone (**2h**)

Off-white solid, yield: 76%; m.p. 39–42°C. ^1^H-NMR (400 MHz, CDCl_3_, *δ*/ppm): 8.35 (1H, s, arom H), 7.78–7.88 (2H, m, arom H), 7.40 (1H, t, *J* = 8.0 Hz, arom H), 4.68 (2H, s, –CH_2_); MS (ESI): *m/z* 233.12 [M^+∙^+H, ^79^Br ^35^Cl], 235.1 [M^+∙^+H+2, ^79^Br ^37^Cl or ^81^Br ^35^Cl], 237.20 [M^+∙^+H+4, ^81^Br, ^37^Cl].

#### 4.5.9. 2-Bromo-1-(4-chlorophenyl)ethanone (**2i**)

Off-white solid, yield: 92%, m.p. 94–96°C. FT-IR (KBr, cm^−1^) 3000.9, 2947.9, 1689.8, 1625.5, 1384.8, 1267.5, 853.3, 679.8, 564.8. ^1^H-NMR (400 MHz, CDCl_3_, *δ*/ppm): 8.12 (2H, d, *J* = 8.0 Hz, arom H), 7.72 (2H, d, *J* = 8.0 Hz, arom H), 4.52 (2H, s, –CH_2_); MS (ESI): *m/z* 233.01 [M^+∙^+H, ^79^Br ^35^Cl], 234.90 [M^+∙^+H+2, ^79^Br ^37^Cl or ^81^Br ^35^Cl], 237.00 [M^+∙^+H+4, ^81^Br, ^37^Cl].

#### 4.5.10. 2-Bromo-1-(4-bromophenyl)ethanone (**2j**)

Off-white solid,yield: 87%; m.p. 108–110°C; ^1^H-NMR (400 MHz, CDCl_3_, *δ*/ppm): 7.90 (2H, d, *J* = 7.6 Hz, arom H), 7.64 (2H, d, *J* = 7.2 Hz, arom H), 4.48 (2H, s, –CH_2_); MS (ESI): *m/z* 278.1 [M^+∙^+H, 2 ^79^Br], 279.88 [M^+∙^+H+2, ^79^Br ^81^Br], 282.02 [M^+∙^+H+4, 2 ^81^Br].

#### 4.5.11. 2-Bromo-1-(4-methoxyphenyl)ethanone (**2k**)

Off-white solid, yield: 72%; m.p. 70–72%; FT-IR (KBr, cm^−1^): 3098, 2938.2, 1688.8, 1600, 1509.1, 1326.2, 1263.7, 1021.4, 817.8, 687.3, 557.8; ^1^H-NMR (400 MHz, CDCl_3_, *δ*/ppm): 7.95 (2H, d, *J* = 7.4 Hz, arom H), 6.95 (2H, d, *J* = 7.4 Hz, arom H), 4.40 (2H, s, –CH_2_), 3.90 (3H, s, –CH_3_). MS (ESI) *m/z* 229.0 [M^+∙^+H, ^79^Br], 231.01 [M^+∙^+H+2, ^81^Br].

#### 4.5.12. 2-Bromo-1-(3-nitrophenyl)ethanone (**2l**)

Yellowish solid, yield: 41%; m.p. 93–96°C; FT-IR (KBr, cm^−1^): 3049, 2947, 1669, 1605.5, 1489.4, 1159.5, 929.6, 853.1, 679.3, 564.5; ^1^H-NMR (400 MHz, CDCl_3_, *δ*/ppm): 8.70 (1H, s, arom H), 8.35–8.55 (2H, m, arom H), 7.80–7.90 (1H, m, arom H), 4.40 (2H, s, –CH_2_); MS (ESI): *m/z* 244.05 [M^+∙^+H, ^79^Br], 246.12 [M^+∙^+H+2, ^81^Br].

#### 4.5.13. 2-Bromo-1-(4-nitrophenyl)ethanone (**2m**)

Yellowish solid, yield: 49%; m.p. 99–102°C; FT-IR (KBr, cm^−1^) 3088, 2992, 1690, 1523, 1347, 1195, 813.2, 729, 618, 490; ^1^H-NMR (400 MHz, CDCl_3_, *δ*/ppm): 8.21 (2H, d, *J* = 7.2 Hz, arom H), 8.61 (2H, d, *J* = 7.2 Hz, arom H), 4.41 (2H, s, –CH_2_); MS (ESI): *m/z* 244.1 [M^+∙^+H, ^79^Br], 245.97 [M^+∙^+H+2, ^81^Br].

#### 4.5.14. 2-Bromo-1-(naphthalen-1-yl)ethanone (**2n**)

Brownish liquid, yield: 84%; b.p. 348.5°C; FT-IR (KBr, cm^−1^): 3049.9, 2948, 1689.8, 1625.4, 1469.3, 1174.6, 1126.9, 1029.5, 853.3, 811.6, 564.7; ^1^H-NMR (400 MHz, CDCl_3_, *δ*/ppm): 8.75 (1H, d, *J* = 7.2 Hz, arom H), 7.95–8.19 (4H, m, arom H), 7.65–7.75 (2H, m, arom H), 4.70 (2H, s, –CH_2_); MS (ESI): *m/z* 249.0 [M^+∙^+H, ^79^Br], 251.1 [M^+∙^+H+2, ^81^Br].

#### 4.5.15. 2-Bromo-1-(naphthalen-2-yl)ethanone (**2o**)

Pale yellowish solid, yield: 91%; m.p. 81–83°C; FT-IR (KBr, cm^−1^): 3088.2, 2997.4, 1702.4, 1610.2, 1434.7, 1199.8, 1082.9, 873.4, 818.2, 739.3, 618.8; ^1^H-NMR (400 MHz, CDCl_3_, *δ*/ppm): 8.80 (1H, d, *J* = 7.6 Hz, arom H), 7.95–8.19 (4H, m, arom H), 7.60–7.78 (2H, m, arom H), 4.30 (2H, s, –CH_2_); MS (ESI): *m/z* 249.1 [M^+∙^+H, ^79^Br], 251.05 [M^+∙^+H+2, ^81^Br].

#### 4.5.16.,2-Dibromo-1-phenylethanone (**3a**)

Colorless liquid, yield: 8–10%; ^1^H-NMR (400 MHz, CDCl_3_, *δ*/ppm): 8.1–8.15 (2H, m, arom H), 7.51–7.81 (3H, m, arom H), 6.60 (1H, s, –CH); MS (ESI): *m/z* 277.03 [M^+∙^+H, 2 ^79^Br], 278.920 [M^+∙^+H+2, ^79^Br, ^81^Br], 281.01 [M^+∙^+H+4, ^81^Br].

#### 4.5.17. 1-(3-Bromo-4-hydroxyphenyl)ethanone (**4a**)

Off-white solid, yield: 94%; ^1^H-NMR (400 MHz, CDCl_3_, *δ*/ppm): 8.18 (1H, s, arom H), 7.95 (1H, d, *J* = 7.2 Hz, arom H), 7.22 (1H, d, *J* = 7.2 Hz, arom H), 5.42 (1H, s, OH), 2.58 (3H, s, –CH_3_); MS (ESI): *m/z* 215.1 [M^+∙^+H, ^79^Br], 217.08 [M^+∙^+H+2, ^81^Br].

#### 4.5.18. 1-(5-Bromo-2-hydroxyphenyl)ethanone (**4b**)

Off-white solid, yield: 68%; m.p. 43–45°C; ^1^H-NMR (400 MHz, CDCl_3_, *δ*/ppm): 12.10 (1H, s, –OH), 8.2 (1H, s, arom H), 8.02 (1H, d, *J* = 7.2 Hz, arom H), 7.22 (1H, d, *J* = 7.6 Hz, arom H), 2.62 (3H, s, –CH_3_); MS (ESI): *m/z* 215.21 [M^+∙^+H, ^79^Br], 216.94 [M^+∙^+H+2, ^81^Br]

#### 4.5.19. 1-(2-Amino-5-bromophenyl)ethanone (**4c**)

Off-white solid, yield: 72%; m.p. 83–85°C; ^1^H-NMR (400 MHz, CDCl_3_, *δ*/ppm): 8.03 (1H, s, arom H), 7.58 (1H, d, *J* = 6.8 Hz, arom H), 7.13 (1H, d, *J* = 7.2 Hz, arom H), 6.42 (2H, s, –NH_2_), 2.7 (3H, s, –CH_3_); MS (ESI): *m/z* 214.09 [M^+∙^+H, ^79^Br], 215.89 [M^+∙^+H+2, ^81^Br].

#### 4.5.20. 1-(4-Amino-5-bromophenyl)ethanone (**4d**)

Off-white solid, yield: 94%; m.p. 155–157°C; ^1^H-NMR (400 MHz, CDCl_3_, *δ*/ppm): 8.15 (1H, s, arom H); 7.82 (1H, d, *J* = 7.2 Hz, arom H), 6.98 (1H, d, *J* = 7.2 Hz, arom H), 6.23 (2H, bs, –NH_2_), 2.7 (3H, s, –CH_3_); MS (ESI): *m/z* 214.1 [M^+∙^+H, ^79^Br], 215.89 [M^+∙^+H+2, ^81^Br].

#### 4.5.21. 1-(3-Bromo-4-methoxyphenyl)ethanone (**4e**)

Off-white solid, yield: 91%; m.p. 70–72°C; FT-IR (KBr, cm^−1^): 3098, 2938.2, 2840, 1688.8, 1600, 1509.1, 1326.2, 1263.7, 1207.5, 1168.3, 1021.4, 940.8, 817.8, 687.3, 580.1, 557.8; ^1^H-NMR (400 MHz, CDCl_3_, *δ*/ppm): 8.1 (1H, s, arom H), 7.83 (1H, d, *J* = 7.6 Hz, arom H), 6.95 (1H, d, *J* = 7.2 Hz, arom H), 4.01 (3H, s, –CH_3_), 2.71 (3H, s, –CH_3_); MS (ESI) *m/z*: 229.0 [M^+∙^+H, ^79^Br], 231.06 [M^+∙^+H+2, ^81^Br].

#### 4.5.22. 1-(3,5-Bis(benzyloxy)-2-bromophenyl)ethanone (**4f**)

Off-white solid, yield: 98%; m.p. 83–85°C; ^1^H-NMR (400 MHz, CDCl_3_, *δ*/ppm): 7.45–7.25 (10H, m, arom H), 7.59 (1H, s, arom H), 7.68 (1H, s, arom H), 5.02 (2H, s, –CH_2_), 5.14 (2H, s, –CH_2_), 2.6 (3H, s, –CH_3_); MS (ESI): *m/z* 411.00 [M^+∙^+H, ^79^Br], 412.93 [M^+∙^+H+2, ^81^Br].

#### 4.5.23. 1-(3,5-Dibromo-4-hydroxyphenyl)ethanone (**5a**)

Off-white solid, yield: 99%; m.p. 184–188°C; ^1^H-NMR (400 MHz, CDCl_3_, *δ*/ppm): 9.86 (1H, s, –OH), 8.10 (2H, s, arom H), 2.60 (3H, s, –CH_3_); MS (ESI): *m/z* 293.01 [M^+∙^+H, 2 ^79^Br], 294.89 [M^+∙^+H+2, ^79^Br ^81^Br], 297.09 [M^+∙^+H +4, 2 ^81^Br].

#### 4.5.24. 1-(2-Amino-3,5-dibromophenyl)ethanone (**5b**)

Off-white solid, yield: 98%; m.p. 121–123°C; ^1^H-NMR (400 MHz, CDCl_3_, *δ*/ppm): 7.81 (1H, s, arom H), 7.69 (1H, s, arom H), 6.82 (2H, bs, –NH_2_), 2.59 (3H, s, –CH_3_); MS (ESI): *m/z* 293.01 [M^+∙^+H, 2 ^79^Br], 294.89 [M^+∙^+H+2, ^79^Br ^81^Br], 297.1 [M^+∙^+H+4, 2 ^81^Br].

## Supplementary Material

Supplementary information (^1^H NMR, and mass spectral data of compounds) associated with this article can be found in the online version.

## Figures and Tables

**Table 1:** Exploring of catalyst and other reaction conditions for *α*-bromination of **1a** using NBS^a^.

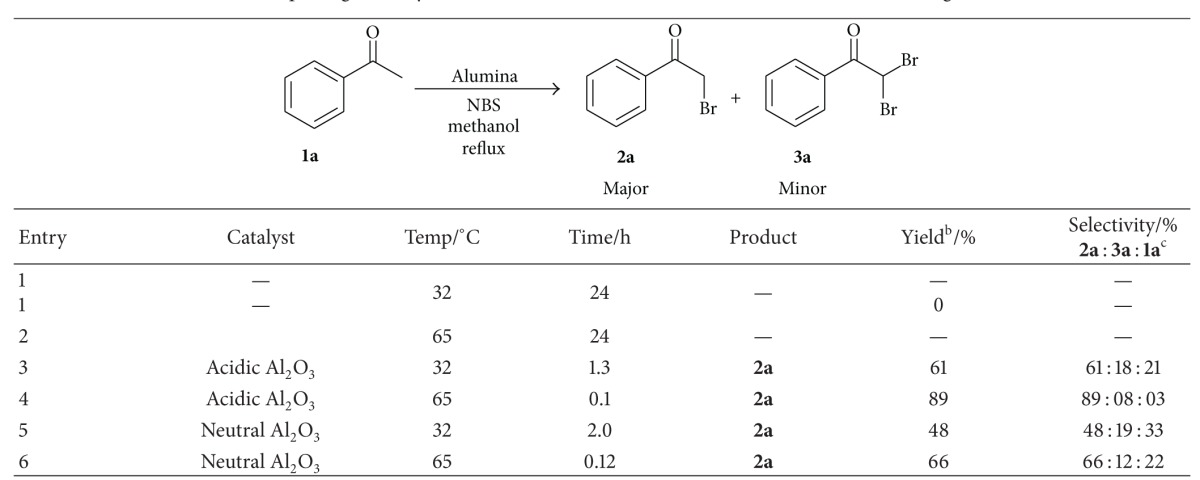

^a^Reaction conditions: acetophenone **1a** (10 mmol), *N*-bromosuccinimide (12 mmol), 10% (w/w) catalyst, and methanol (20 vol) at reflux temperature.

^b^Isolated yield of product **2a**.

^c^Unreacted acetophenone.

**Table 2:** Effect of solvent on *α*-bromination of acetophenone^a^.

Entry	Solvent	Time/min	Product	Yield^b^/%
1	MeOH	10	**2a**	89
2	EtOH	40	**2a**	74
3	H_2_O	24 hrs	**2a**	15
4	CH_3_CN	60	**2a**	51
^ c^5	^ d^Ether	30	**2a**	56
6	^ d^THF	65	**2a**	44
7	CH_2_Cl_2_	180	**2a**	55
8	CHCl_3_	120	**2a**	48

^a^Reaction conditions: acetophenone **1a** (10 mmol), *N*-bromosuccinimide (12 mmol), 10% (w/w) acidic Al_2_O_3_, and solvent (20 vol) at reflux temperature.

^b^Isolated yield.

^c^Reaction was conducted at 32°C.

^d^Freshly distilled ether and THF were used (peroxide free).

**Table 3:** *α*-Bromination of acetophenone derivative(s) containing moderate activating/deactivating or high deactivating groups^a^.

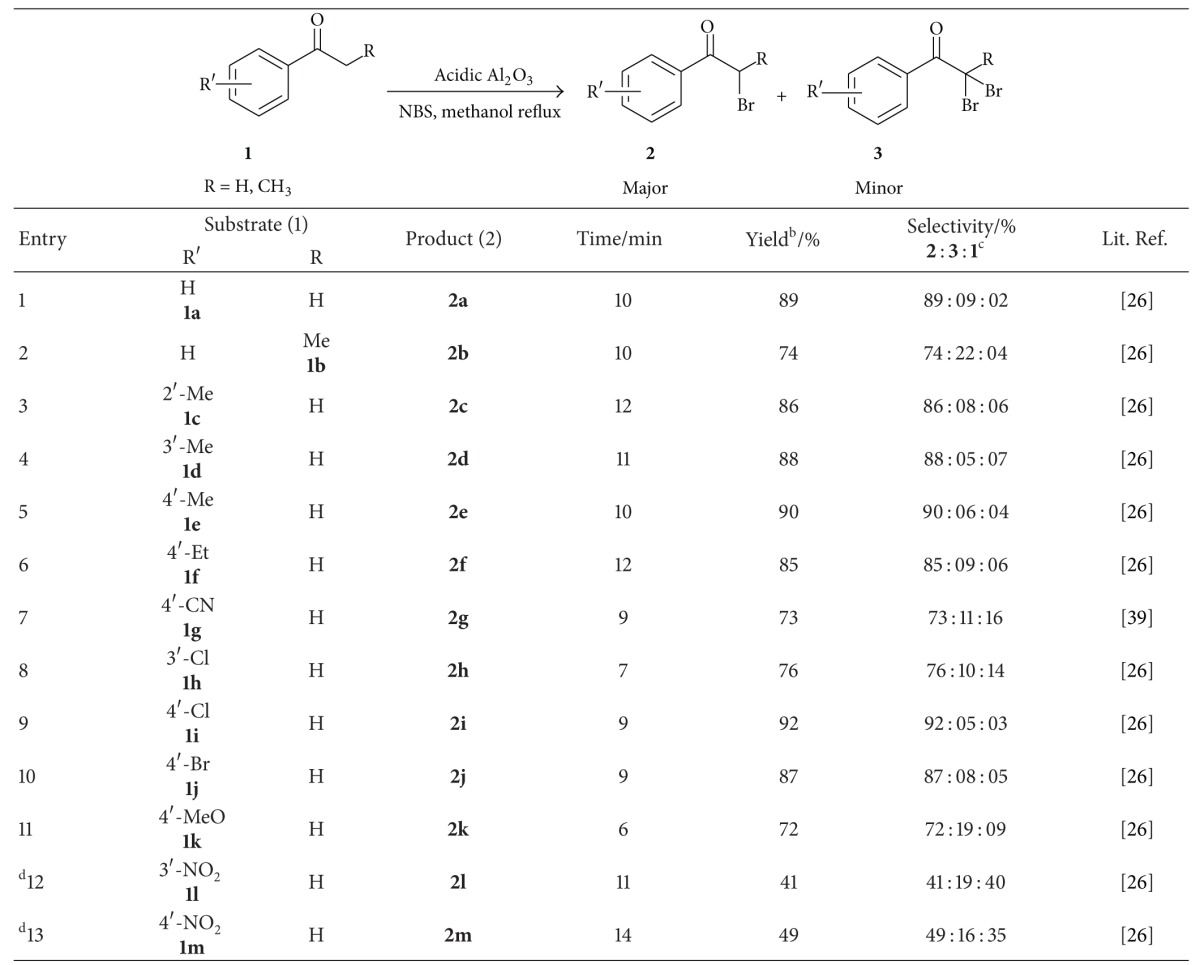

^a^Reaction conditions: **1** (10 mmol), *N*-bromosuccinimide (12 mmol), and 10% (w/w) acidic Al_2_O_3_ in methanol (20 mL) at reflux temperature.

^b^Isolated yield.

^c^Unreacted substrate.

^d^5% acidic Al_2_O_3 _was applied.

**Table 4:** *α*-Bromination of acenaphthone^a^.

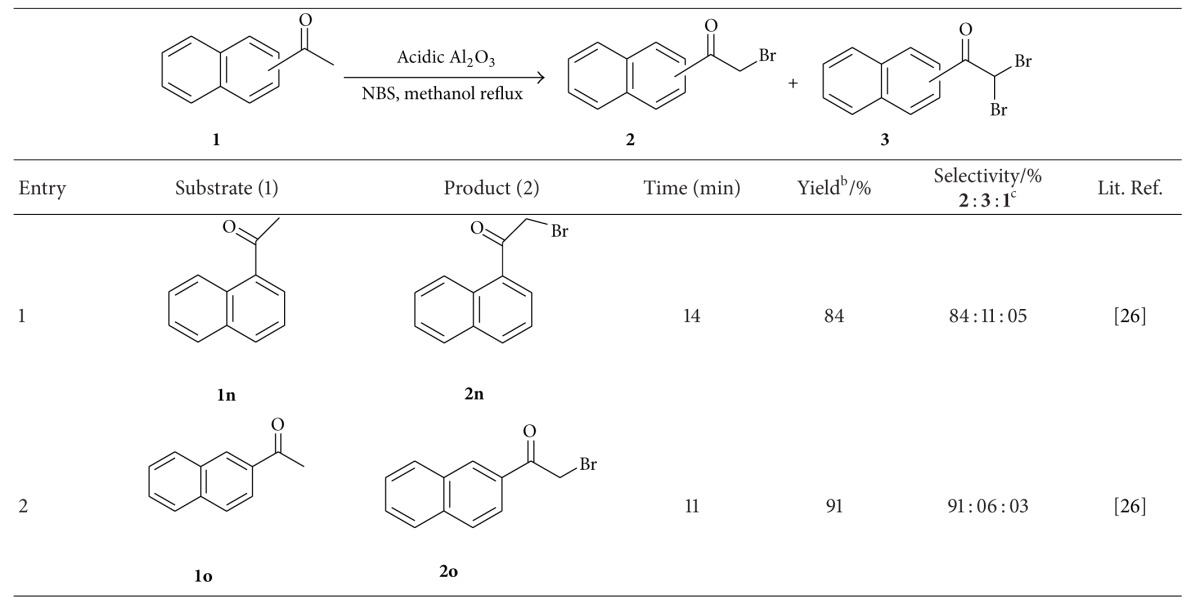

^a^Reaction conditions: acenaphthone **1** (10 mmol), *N*-bromosuccinimide (12 mmol), and 10% acidic Al_2_O_3_ in methanol (20 mL) at reflux temperature.

^b^Isolated yield of desired product(s).

^c^Unreacted substrate.

**Table 5:** Exploring of suitable catalyst for nuclear bromination^a^.

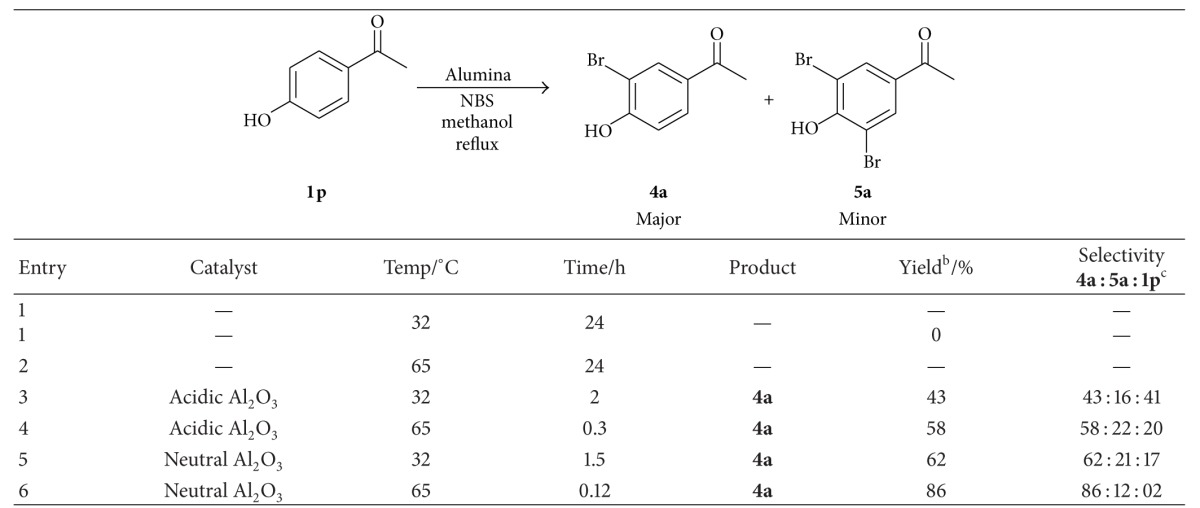

^a^Reaction conditions: 4′-hydroxy acetophenone **1p** (10 mmol), *N*-bromosuccinimide (12 mmol), 10% (w/w) catalyst, and methanol (20 mL) at reflux temperature.

^b^Isolated yield.

^c^Unreacted 4′-hydroxy acetophenone (**1p**).

**Table 6:** Effect of solvent on nuclear bromination of 4′-Hydroxy acetophenone (**1p**)^a^.

Entry	Solvent	Time/min	Product	Yield^b^/%
1	MeOH	12	**4a**	86
2	EtOH	60	**4a**	61
3	H_2_O	24 hrs	**4a**	22
4	CH_3_CN	14	**4a**	94
^ c^5	^ d^Ether	45	**4a**	48
6	^ d^THF	75	**4a**	40
7	CH_2_Cl_2_	150	**4a**	30
8	CHCl_3_	90	**4a**	34

^a^Reaction conditions: 4′-hydroxy acetophenone **1p** (10 mmol), *N*-bromosuccinimide (12 mmol), 10% (w/w) neutral Al_2_O_3_, and solvent (20 vol) at reflux temperature.

^b^Isolated yield.

^c^Reaction was conducted at 32°C.

^d^Freshly distilled ether and THF were used (peroxide free).

**Table 7:** Nuclear bromination of various aralkyl ketones containing high activating groups^a^.

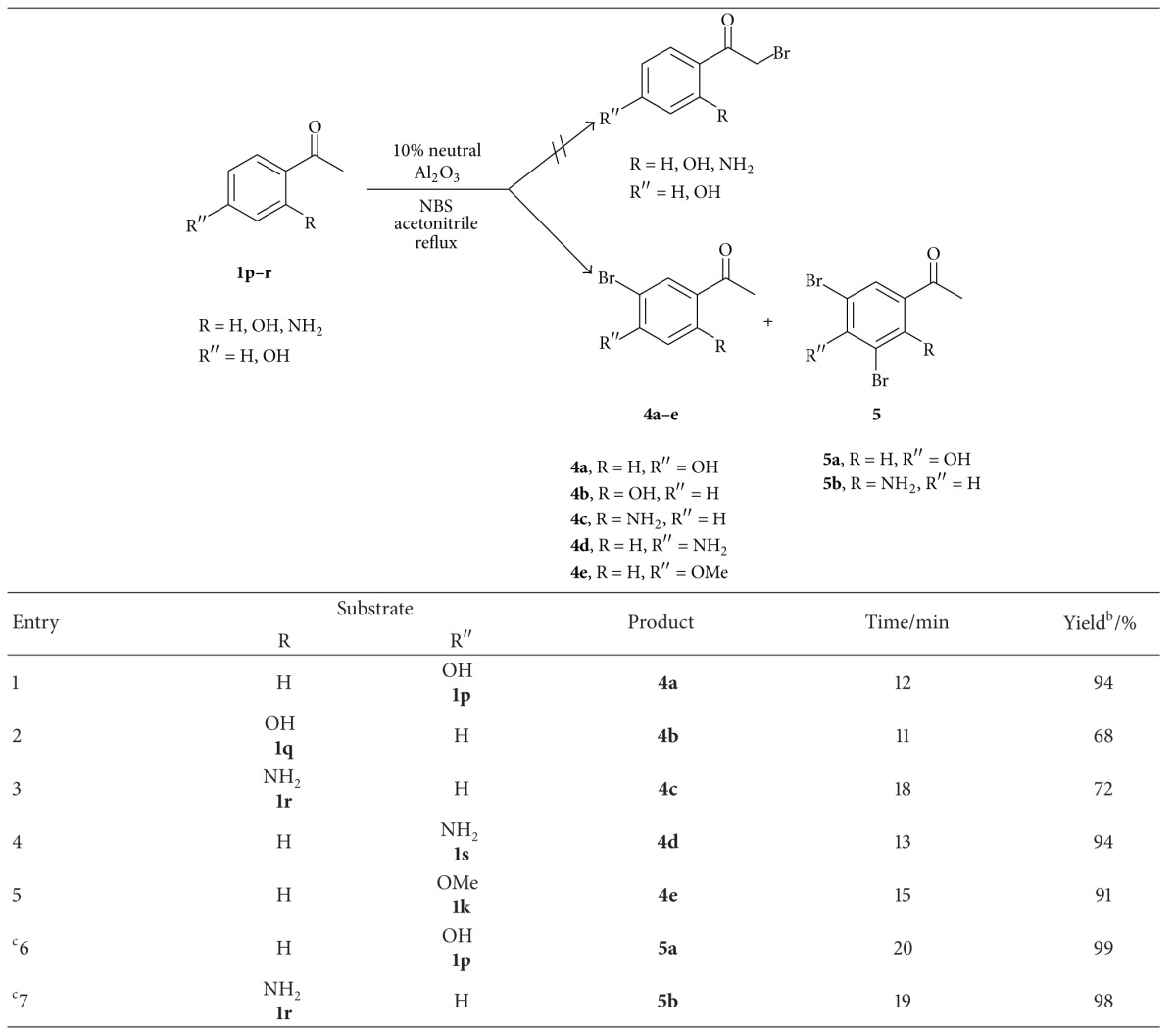

^a^Reaction conditions: aralkyl ketone(s) **1p–r** (10 mmol), *N*-bromosuccinimide (12 mmol), and 10% (w/w) neutral Al_2_O_3_ in acetonitrile (20 mL) at reflux temperature.

^b^Isolated yield.

^c^22 mmol of NBS was utilized.
