# Supplementary material for: Substrate Directed Regioselective Monobromination of Aralkyl Ketones Using N-Bromosuccinimide Catalysed by Active Aluminium Oxide: α-Bromination versus Ring Bromination
Source: ISRN Org Chem. 2014 Mar 4;2014:751298. doi: 10.1155/2014/751298 (PMC4041026; doi:10.1155/2014/751298)
Supplement: Supplementary file 1 — Supplementary information (1H NMR, and mass spectral data of compounds) associated with this article can be found in the online version. [file 751298.f1.doc]

**Supplementary Material**

**Substrate Directed Regioselective Monobromination of Aralkyl Ketones Using** *N***-Bromosuccinimide Catalysed by Active Aluminium Oxide: 𝛼-Bromination versus Ring Bromination**

**Reddy Bodireddy Mohan, G. Trivikram Reddy, and N. C. Gangi Reddy**

*Department of Chemistry, School of Physical Sciences, Yogi Vemana University, Kadapa, Andhra Pradesh 516 003, India*

*E-mail; ncgreddy@yogivemanauniversity.ac.in, Phone:+91-8562-225410, Mobile:+91-9966715535*

**Physical and Spectral Characterization Data of compounds** (**2a-2o**): The physical and spectral characterization data of the synthesized 𝛼-brominated products (**2a-2o**) are given below

**2-Bromo-1-phenyl ethanone (2a)**^1^ **[Table 3, entry 1]:**

Off-white solid, yield: 89%; m.p. 48–50°C; FT-IR (KBr, cm^-1^): 3085.6, 2999.6, 1694.3, 1589.7, 1485.8, 1283.6, 1198.8, 811.8, 665.7, 548.1; ^1^HNMR (400MHz,CDCl_3_, 𝛿/ppm): 8.00–8.10 (2H,m, aromH), 7.43–7.78 (3H, m, arom H), 4.50 (2H, s, –CH_2_). MS (ESI) *m/z* 199.05 [M^+^**^∙^**+H, ^79^Br], 201.1 [M^+^**^∙^**+H +2, ^81^Br].

**2-Bromo-1-phenylpropan-1-one (2b)**^1^ **[Table 3, entry 2]:**

Colorless liquid, yield: 74%; b.p. 247–251°C; ^1^HNMR (400MHz,CDCl_3_, 𝛿/ppm): 8.10 (2H, d, *J* = 7.2Hz, arom H), 7.72–7.64 (1H, m, arom H), 7.50-7.60 (2H, m, arom H), 5.30 (1H, q, *J* = 6.8Hz, alkyl H), 2.10 (3H, d, *J* = 7.2Hz, –CH_3_); MS (ESI) *m/z* 213.03 [M^+^**^∙^**+H, ^79^Br], 215.2 [M^+^**^∙^**+H+2, ^81^Br].

**2-Bromo-1-o-tolylethanone (2c)**^1^ **[Table 3, entry 3]:**

Colorless liquid, yield: 86%; b.p. 81–83°C; ^1^HNMR (400MHz,CDCl_3_, 𝛿/ppm): 8.10 (1H, d, arom H, *J* = 8.0Hz), 7.70 (1H, t, *J* = 7.6Hz, arom H), 7.40 (1H, t, *J* = 7.2Hz, arom H), 7.10 (1H, d, *J* =8.0Hz, arom H), 4.50 (2H, s, –CH_2_), 2.50 (3H, s, –CH_3_); MS (ESI): *m/z* 212.96 [M^+^**^∙^**+H, ^79^Br], 214.97 [M^+^**^∙^**+H+2, ^81^Br].

**2-Bromo-1-m-tolylethanone (2d)**^1^ **[Table 3, entry 4]:**

Colorless liquid, yield: 88%; b.p. 234°C; ^1^HNMR (400MHz,CDCl_3_, 𝛿/ppm): 7.83 (1H, d, *J* = 8.0Hz, arom H), 7.58 (1H, s, arom H), 7.35 (1H, d, *J* = 7.6Hz, arom H), 7.10 (1H, t, *J* = 7.2Hz, arom H), 4.60 (2H, s, –CH_2_), 2.48 (3H, s, –CH_3_); MS (ESI): *m/z* 213.15 [M^+^**^∙^**+H, ^79^Br], 215.1 [M^+^**^∙^**+H+2, ^81^Br].

**2-Bromo-1-p-tolylethanone (2e)**^1^ **[Table 3, entry 5]:**

Off-white solid, yield: 90%, m.p. 51–53°C. ^1^HNMR (400MHz,CDCl_3_, 𝛿/ppm): 7.65 (2H, d, *J* = 7.6Hz, arom H), 7.00 (2H, d, *J* = 7.2Hz, aromH), 4.70 (2H, s, –CH_2_), 2.54 (3H, s, –CH_3_); MS (ESI): *m/z* 212.89 [M^+^**^∙^**+H, ^79^Br], 214.81 [M^+^**^∙^**+H +2, ^81^Br].

**2-Bromo-1-(4-ethylphenyl)ethanone (2f)**^1^ **[Table 3, entry 6]:**

Colorless liquid, yield: 85%; b.p. 288.5°C. ^1^HNMR (400MHz,CDCl_3_, 𝛿/ppm): 7.45 (2H, d, *J* = 8.0Hz, arom H), 7.20 (2H, d, *J* = 7.6Hz, aromH), 4.60 (2H, s, –CH_2_), 2.64 (2H, q, *J* = 7.6Hz, –CH_2_), 1.54 (3H, t, *J* = 6.8Hz, –CH_3_); MS (ESI): *m/z* 226.95 [M^+^**^∙^**+H, ^79^Br], 229.08 [M^+^**^∙^**+H+2, ^81^Br].

**4-(2-Bromoacetyl)benzonitrile (2g)**^1^ **[Table 3, entry 7]:**

Off-white solid, yield: 73%, m.p. 90–92°C. ^1^HNMR (400MHz,CDCl_3_, 𝛿/ppm): 8.24 (2H, d, *J* = 7.2Hz, aromH), 7.91 (2H, d, *J* =7.6Hz, arom H), 4.32 (2H, s, –CH_2_); MS (ESI): *m/z* 224.15 [M^+^**^∙^**+H, ^79^Br], 226.01 [M^+^**^∙^**+H+2, ^81^Br].

**2-Bromo-1-(3-chlorophenyl)ethanone (2h)**^1^ **[Table 3, entry 8]:**

Off-white solid, yield: 76%; m.p. 39–42°C. ^1^HNMR (400MHz,CDCl_3_, 𝛿/ppm): 8.35 (1H, s, aromH), 7.78–7.88 (2H, m, aromH), 7.40 (1H, t, *J* = 8.0Hz, aromH), 4.68 (2H, s, –CH_2_); MS (ESI): *m/z* 233.12 [M^+^**^∙^**+H, ^79^Br ^35^Cl], 235.1 [M^+^**^∙^**+H+2, ^79^Br ^37^Cl or ^81^Br ^35^Cl], 237.20 [M^+^**^∙^**+H+4, ^81^Br, ^37^Cl].

**2-Bromo-1-(4-chlorophenyl)ethanone (2i)**^1^ **[Table 3, entry 9]:**

Off-white solid, yield: 92%, m.p. 94–96°C. FT-IR (KBr, cm^-1^) 3000.9, 2947.9, 1689.8, 1625.5, 1384.8, 1267.5, 853.3, 679.8, 564.8.; ^1^HNMR (400MHz,CDCl_3_, 𝛿/ppm): 8.12 (2H, d, *J* = 8.0Hz, arom H), 7.72 (2H, d, *J* = 8.0Hz, aromH), 4.52 (2H, s, –CH_2_); MS (ESI): *m/z* 233.01 [M^+^**^∙^**+H, ^79^Br ^35^Cl], 234.90 [M^+^**^∙^**+H+2, ^79^Br ^37^Cl or ^81^Br ^35^Cl], 237.00 [M^+^**^∙^**+H+4, ^81^Br, ^37^Cl].

**2-Bromo-1-(4-bromophenyl)ethanone (2j)**^1^ **[Table 3, entry 10]:**

Off-white solid, yield: 87%; m.p. 108–110°C; ^1^HNMR (400MHz,CDCl_3_, 𝛿/ppm): 7.90 (2H, d, *J* = 7.6Hz, arom H), 7.64 (2H, d, *J* = 7.2Hz, arom H), 4.48 (2H, s, –CH_2_); MS (ESI): *m/z* 278.1 [M^+^**^∙^**+H, 2^79^Br], 279.88 [M^+^**^∙^**+H+2, ^79^Br ^81^Br], 282.02 [M^+^**^∙^**+H+4, 2^81^Br].

**2-Bromo-1-(4-methoxyphenyl)ethanone (2k)**^1^ **[Table 3, entry 11]:**

Off-white solid, yield: 72%; m.p. 70–72°C; FT-IR (KBr, cm^-1^): 3098, 2938.2, 1688.8, 1600, 1509.1, 1326.2, 1263.7, 1021.4, 817.8, 687.3, 557.8; ^1^HNMR (400MHz,CDCl_3_, 𝛿/ppm): 7.95 (2H, d, *J* = 7.4Hz, arom H), 6.95 (2H, d, *J* = 7.4Hz, arom H), 4.40 (2H, s, –CH_2_), 3.90 (3H, s, –CH_3_). MS (ESI) *m/z* 229.0 [M^+^**^∙^**+H, ^79^Br], 231.01 [M^+^**^∙^**+H+2, ^81^Br].

**2-Bromo-1-(3-nitrophenyl)ethanone (2l)**^1^ **[Table 3, entry 12]:**

Yellowish solid, yield: 41%; m.p. 93–96°C; FT-IR (KBr, cm^-1^): 3049, 2947, 1669, 1605.5, 1489.4, 1159.5, 929.6, 853.1, 679.3, 564.5; ^1^H-NMR (400MHz, CDCl_3_, 𝛿/ppm): 8.70 (1H, s, arom H), 8.35–8.55 (2H, m, arom H), 7.80-7.90 (1H, m, arom H), 4.40 (2H, s, –CH_2_); MS (ESI): *m/z* 244.05 [M^+^**^∙^**+H, ^79^Br], 246.12 [M^+^**^∙^**+H+2, ^81^Br].

**2-Bromo-1-(4-nitrophenyl)ethanone (2m)**^1^ **[Table 3, entry 13]:**

Yellowish solid, yield: 49%; m.p. 99–102°C; FT-IR (KBr, cm^-1^) 3088, 2992, 1690, 1523, 1347, 1195, 813.2, 729, 618, 490; ^1^HNMR (400MHz,CDCl_3_, 𝛿/ppm): 8.21 (2H, d, *J* = 7.2Hz, aromH), 8.61 (2H, d, *J* = 7.2Hz, aromH), 4.41 (2H, s, –CH_2_); MS (ESI): *m/z* 244.1 [M^+^**^∙^**+H, ^79^Br], 245.97 [M^+^**^∙^**+H+2, ^81^Br].

**2-Bromo-1-(naphthalen-1-yl)ethanone (2n)**^1^ **[Table 4, entry 1]:**

Brownish liquid, yield: 84%; b.p. 348.5°C; FT-IR (KBr, cm^-1^): 3049.9, 2948, 1689.8, 1625.4, 1469.3, 1174.6, 1126.9, 1029.5, 853.3, 811.6, 564.7; ^1^HNMR (400MHz,CDCl_3_, 𝛿/ppm): 8.75 (1H, d, *J* = 7.2Hz, arom H), 7.95–8.19 (4H, m, aromH), 7.65-7.75 (2H, m, arom H), 4.70 (2H, s, –CH_2_); MS (ESI): *m/z* 249.0 [M^+^**^∙^**+H, 79Br], 251.1 [M^+^**^∙^**+H+2, ^81^Br].

**2-Bromo-1-(naphthalen-2-yl)ethanone (2o)**^1^ **[Table 4, entry 2]:**

Pale yellowish solid, yield: 91%; m.p. 81–83°C; FT-IR (KBr, cm^-1^): 3088.2, 2997.4, 1702.4, 1610.2, 1434.7, 1199.8, 1082.9, 873.4, 818.2, 739.3, 618.8; ^1^HNMR (400MHz,CDCl_3_, 𝛿/ppm): 8.80 (1H, d, *J* = 7.6Hz, arom H), 7.95–8.19 (4H, m, arom H), 7.60–7.78 (2H, m, aromH), 4.30 (2H, s, –CH_2_); MS (ESI): *m/z* 249.1 [M^+^**^∙^**+H, ^79^Br], 251.05 [M^+^**^∙^**+H+2, ^81^Br].

**Physical and Spectral Characterization Data of compound 3a**: The physical and spectral characterization data of the synthesized 𝛼-dibrominated product (**3a**) is given below

**2,2-Dibromo-1-phenylethanone (3a)** **[Table 3, entry 1]:**

Colorless liquid, yield: 8–10%; ^1^HNMR (400MHz,CDCl_3_, 𝛿/ppm): 8.1–8.15 (2H, m, aromH), 7.51–7.81 (3H, m, arom H), 6.60 (1H, s, –CH); MS (ESI): *m/z* 277.03 [M^+^**^∙^**+H, 2 ^79^Br], 278.920 [M^+^**^∙^**+H +2, ^79^Br, ^81^Br], 281.01 [M^+^**^∙^**+H +4, ^81^Br].

**Physical and Spectral Characterization Data of compounds** (**4a-4f**): The physical and spectral characterization data of the synthesized ring brominated products (**4a-4f**) are given below

**1-(3-Bromo-4-hydroxyphenyl)ethanone (4a)**^2,3^ **[Table 7, entry 1]:**

Off-white solid, yield: 94%; ^1^HNMR (400MHz,CDCl_3_, 𝛿/ppm): 8.18 (1H, s, aromH), 7.95 (1H, d, *J* = 7.2Hz, aromH), 7.22 (1H, d, *J* = 7.2Hz, arom H), 5.42 (1H, s, OH), 2.58 (3H, s, –CH_3_); MS (ESI): *m/z* 215.1 [M^+^**^∙^**+H, ^79^Br], 217.08 [M^+^**^∙^**+H+2, ^81^Br].

**1-(5-Bromo-2-hydroxyphenyl)ethanone (4b)**^4^ **[Table 7, entry 2]:**

Off-white solid, yield: 68%; m.p. 43–45°C; ^1^HNMR (400MHz,CDCl_3_, 𝛿/ppm): 12.10 (1H, s, –OH), 8.2 (1H, s, arom H), 8.02 (1H, d, *J* = 7.2Hz, arom H), 7.22 (1H, d, *J* = 7.6Hz, arom H), 2.62 (3H, s, –CH_3_); MS (ESI): *m/z* 215.21 [M^+^**^∙^**+H, ^79^Br], 216.94 [M^+^**^∙^**+H+2, ^81^Br].

**1-(2-Amino-5-bromophenyl)ethanone (4c)**^5^ **[Table 7, entry 3]:**

Off-white solid, yield: 72%; m.p. 83–85°C; ^1^HNMR (400MHz,CDCl_3_, 𝛿/ppm): 8.03 (1H, s, arom H), 7.58 (1H, d, *J* = 6.8Hz, arom H), 7.13 (1H, d, *J* = 7.2Hz, arom H), 6.42 (2H, s, –NH_2_), 2.7 (3H, s, –CH_3_); MS (ESI): *m/z* 214.09 [M^+^**^∙^**+H, ^79^Br], 215.89 [M^+^**^∙^**+H+2, ^81^Br].

**1-(4-Amino-5-bromophenyl)ethanone (4d) [Table 7, entry 4]:**

Off-white solid, yield: 94%; m.p. 155–157°C; ^1^H-NMR (400MHz, CDCl_3_, 𝛿/ppm): 8.15 (1H, s, arom H); 7.82 (1H, d, *J* = 7.2Hz, arom H), 6.98 (1H, d, *J* = 7.2Hz, arom H), 6.23 (2H, bs, –NH_2_), 2.7 (3H, s, –CH_3_); MS (ESI): *m/z* 214.1 [M^+^**^∙^**+H, ^79^Br], 215.89 [M^+^**^∙^**+H+2, ^81^Br].

**1-(3-Bromo-4-methoxyphenyl)ethanone (4e) [Table 7, entry 5]:**

Off-white solid, yield: 91%; m.p. 70–72°C; FT-IR (KBr, cm^-1^): 3098, 2938.2, 2840, 1688.8, 1600, 1509.1, 1326.2, 1263.7, 1207.5, 1168.3, 1021.4, 940.8, 817.8, 687.3, 580.1, 557.8; ^1^H-NMR (400MHz, CDCl_3_, 𝛿/ppm): 8.1 (1H, s, arom H), 7.83 (1H, d, *J*= 7.6Hz, arom H), 6.95 (1H, d, *J* = 7.2Hz, arom H), 4.01 (3H, s, –CH_3_), 2.71 (3H, s, –CH_3_); MS (ESI) *m/z*: 229.0 [M^+^**^∙^**+H, ^79^Br], 231.06 [M^+^**^∙^**+H+2, ^81^Br].

**1-(3,5-Bis(benzyloxy)-2-bromophenyl)ethanone(4f) [scheme 4]:**

Off-white solid, yield: 98%; m.p. 83–85°C; ^1^H-NMR (400MHz, CDCl_3_, 𝛿/ppm): 7.45–7.25 (10H, m, arom H), 7.59 (1H, s, arom H), 7.68 (1H, s, arom H), 5.02 (2H, s, –CH_2_), 5.14 (2H, s, –CH_2_), 2.6 (3H, s, –CH_3_); MS (ESI): *m/z* 411.05 [M^+^**^∙^**+H, ^79^Br], 412.93 [M^+^**^∙^**+H+2, ^81^Br].

**Physical and Spectral Characterization Data of compounds** (**5a & 5b**): The physical and spectral characterization data of the synthesized ring dibrominated products (**5a & 5b**) are given below

**1-(3,5-Dibromo-4-hydroxyphenyl)ethanone (5a)**^6^ **[Table 7, entry 6]:**

Off-white solid, yield: 99%; m.p. 184–188°C; ^1^H-NMR (400MHz, CDCl_3_, 𝛿/ppm): 9.86 (1H, s, –OH), 8.10 (2H, s, arom H), 2.60 (3H, s, –CH_3_); MS (ESI): *m/z* 293.01 [M^+^**^∙^**+H, 2^79^Br], 294.89 [M^+^**^∙^**+H+2, ^79^Br ^81^Br], 297.09 [M^+^**^∙^**+H +4, 2^81^Br].

**1-(2-Amino-3,5-dibromophenyl)ethanone (5b) [Table 7, entry 7]:**

Off-white solid, yield: 98%; m.p. 121–123°C; ^1^H-NMR (400MHz, CDCl_3_, 𝛿/ppm): 7.81 (1H, s, arom H), 7.69 (1H, s, arom H), 6.82 (2H, bs, –NH_2_), 2.59 (3H, s, –CH_3_); MS (ESI): *m/z* 293.01 [M^+^**^∙^**+H, 2^79^Br], 294.89 [M^+^**^∙^**+H+2, ^79^Br ^81^Br], 297.1 [M^+^**^∙^**+H+4, 2^81^Br].

**References:** For copies of spectra see

1. R. B. Mohan and N. C. Gangi Reddy, “Regioselective 𝛼-bromination of aralkylketones using *N*-bromosuccinimide in presence of montmorillonite k-10 clay: a simple and efficient method,” *Synthetic Communications*, vol. 43, no. 19, pp. 2603–2614, 2013.
2. D. E. Pearson, H. W. Pope, “The swamping catalyst effect in bromination of acetophenone,” *The* *Journal of Organic Chemistry,* vol. 21, no. 3, pp. 381-381, 1956.
3. N.P. Buu-Hoï, D. Lavit, “The bromination of *o*- and *p*-hydroxyaryl ketones,” *Journal of the Chemical Society*, vol. 0, no. 0, pp.18-20, 1955.
4. A. T. Johnson, L. Wang, A. M. Standeven, M. Escobar, R. A. S. Chandraratna, “Synthesis and biological activity of high-affinity retinoic acid receptor antagonists,” *Bioorganic & Medicinal Chemistry,* Vol. *7*, no. 7, pp.1321-1338, 1999.
5. T. Nittoli, K. Curran, S. Insaf, M. D. Grandi, M. Orlowski, R. Chopra, A. Agarwal, A. Y. M. Howe, A. Prashad, M. B. Floyd, B. Johnson, A. Sutherland, K. Wheless, B. Feld, J. O. Connell, T. S. Mansour, J. Bloom, “Identification of Anthranilic acid derivatives as a novel class of Allosteric inhibitors of Hepatitis C NS5B Polymerase,” *Journal of Medicinal Chem*istry, vol. 50, no. 9, pp.2108-2116, 2007.
6. N. P. Buu-Hoï, D. Lavit, “The bromination of *o*- and *p*-hydroxyaryl ketones,” *Journal of the Chemical Society*, pp.18-20, 1955.
